# Bortezomib Plus Continuous B Cell Depletion Results in Sustained Plasma Cell Depletion and Amelioration of Lupus Nephritis in NZB/W F1 Mice

**DOI:** 10.1371/journal.pone.0135081

**Published:** 2015-08-07

**Authors:** Laleh Khodadadi, Qingyu Cheng, Tobias Alexander, Özen Sercan-Alp, Jens Klotsche, Andreas Radbruch, Falk Hiepe, Bimba F. Hoyer, Adriano Taddeo

**Affiliations:** 1 German Rheumatism Research Center Berlin (DRFZ) - a Leibniz Institute, Berlin, Germany; 2 Department of Rheumatology and Clinical Immunology, Charité University Hospital Berlin, Berlin, Germany; INSERM-Université Paris-Sud, FRANCE

## Abstract

Long-lived plasma cells (LLPCs) are an unmet therapeutic challenge, and developing strategies for their targeting is an emerging goal of autoantibody-mediated diseases such as systemic lupus erythematosus (SLE). It was previously shown that plasma cells can be depleted by agents such as bortezomib (Bz) or by blocking LFA-1 and VLA-4 integrins. However, they regenerate quickly after depletion due to B cell hyperactivity in autoimmune conditions. Therefore, we compared different therapies for the elimination of LLPCs combined with selective B-cell targeting in order to identify the most effective treatment to eliminate LLPCs and prevent their regeneration in lupus-prone NZB/W F1 mice.

**Methods:**

NZB/W F1 mice were treated with: 1) anti-CD20, 2) anti-CD20 plus bortezomib, 3) anti-CD20 plus anti-LFA-1/anti-VLA-4 blocking antibodies, 4) anti-CD20 plus bortezomib and anti-LFA-1/anti-VLA4 blocking antibodies. Short- and long-lived plasma cells including autoreactive cells in the bone marrow and spleen were enumerated by flow cytometry and ELISPOT seven days after treatment. Based on these data in another experiment, mice received one cycle of anti-CD20 plus bortezomib followed by four cycles of anti-CD20 therapy every 10 days and were monitored for its effect on plasma cells and disease.

**Results:**

Short-lived plasma cells in bone marrow and spleen were efficiently depleted by all regimens targeting plasma cells. Conversely, LLPCs and anti-dsDNA-secreting plasma cells in bone marrow and spleen showed resistance to depletion and were strongly reduced by bortezomib plus anti-CD20. The effective depletion of plasma cells by bortezomib complemented by the continuous depletion of their precursor B cells using anti-CD20 promoted the persistent reduction of IgG anti-dsDNA antibodies, delayed nephritis and prolonged survival in NZB/W F1 mice.

**Conclusions:**

These findings suggest that the effective depletion of LLPCs using bortezomib in combination with a therapy that continuously targeting B cells as their precursors may prevent the regeneration of autoreactive LLPCs and, thus, might represent a promising treatment strategy for SLE and other (auto)antibody-mediated diseases.

## Introduction

Aberrant production of autoantibodies against diverse nuclear antigens is a hallmark of systemic lupus erythematosus (SLE) [1, 2]. In 1997 [3] and 1998 [4], two groups independently showed that persistent antibody titers are caused by long-lived plasma cells (LLPCs). These cells, which reside in dedicated survival niches in the bone marrow and spleen, are responsible for the maintenance of “humoral memory”. In 2004, we demonstrated that both short- and long-lived plasma cells significantly contribute to chronic humoral autoimmunity in NZB/W F1 mice, a model of SLE [5]. Our recent study also demonstrated that autoreactive LLPCs are able to induce immune complex nephritis when transferred into immunodeficient Rag-/- mice, critically contributing to autoimmune pathology [6]. While immunosuppressive therapy and anti-CD20 monoclonal antibody (mAb) therapy can deplete short-lived plasmablasts and plasma cells (SLPCs), LLPCs are resistant to immunosuppressive drugs [5, 7] and B-cell depletion (BCD) therapies [8]. These findings indicate that targeting pathogenic LLPCs could be promising for the treatment of SLE patients.

New therapeutic options for targeting of LLPCs have emerged during the past decade [8]. Considering that bone marrow plasma cells express leukocyte function-associated antigen-1 (LFA-1) and very late antigen-4 (VLA-4), these integrins using specific antibodies were blocked to induce the temporary depletion of plasma cells in non-autoimmune mice [9]. Bortezomib (Bz), a selective inhibitor of the 26S proteasome subunit, has been shown to be effective in depleting (short- and long-lived) plasma cells in lupus mice and protecting the mice from nephritis [10]. However, it must be noted that as soon as plasma cell depletion treatment is discontinued, these cells can be quickly replenished by activation of autoreactive B cells, as was recently shown in lupus mice and SLE patients [10–12]. Direct B-cell depletion (BCD), although ineffective in eliminating LLPCs, may interrupt the generation of new autoreactive SLPCs and LLPCs that result from B-cell hyperreactivity [13, 14]. Moreover, BCD might limit the capacity of B cells to promote disease in an antibody-independent manner, representing a useful complement to LLPC depletion.

In this study, we compared the short-term effect of different approaches for targeting LLPCs (bortezomib, and anti-LFA-1 plus anti-VLA-4 blocking antibodies) in combination with a BCD agent (anti-mouse CD20 antibody) to identify the best and most efficient method for initial short-term depletion of these cells. We showed that, in lupus prone NZB/W F1 mice, the proteasome inhibitor bortezomib combined with a B-cell-depleting agent (i.e., anti-CD20-depleting antibody) was the most effective treatment for plasma cell depletion. The substantial depletion of SLPCs and LLPCs together with the targeting of plasma cell precursors by continuous BCD therapy could induce a long-lasting improvement of disease. This preclinical model of combined immunotherapy targeting both plasma cells and their precursors may provide useful information for the development of therapeutic concepts in SLE and other antibody-mediated diseases.

## Methods

### Mice

Female NZB/W F1 mice were bred and maintained in specific pathogen-free conditions at the mouse facility of German Rheumatism Research Centre (DRFZ) in Berlin, Germany. All animal procedures were approved by the local authority for animal research procedures, the State Office of Health and Welfare (LAGeSo) of Berlin, Germany.

### Depletion regimens

The following antibodies were used for treatment: mouse anti-mouse CD20-specific mAbs (clone 18B12, isotype IgG2a, kindly provided by Biogen Idec), anti-VLA-4 (clone PS/2, isotype IgG2b) and anti-LFA-1 (clone M17/4, isotype IgG2a) blocking mAbs were purified from hybridoma (American Type Culture Collection) (DRFZ) and bortezomib (Velcade) was purchased from Millennium Pharmaceuticals. For standardization, the same doses, routes and times of administration of depletive agents were used in the respective groups; anti-CD20 (10 mg/kg IV/day 0), bortezomib (0.75 mg/kg IV/ days 4.5 and 6, 36 h-interval) and co-injection of anti-LFA-1 and anti-VLA-4 (200 μg of each antibody/mouse IP on days 1 and 3).

#### Short-term initial B- and plasma-cell depletion (STD)

To compare the efficiency of different short-term B- and plasma-cell depletion regimens, 20- to 22-week-old NZB/W F1 mice (at the age of mild disease; low proteinuria and high autoantibody titers) were fed bromodeoxyuridine (BrdU) (Sigma-Aldrich) (1 mg/ml) dissolved in drinking water containing 1% glucose for a period of two weeks, starting one week before treatment. Mice were divided into five groups and treated with a) vehicle (phosphate-buffered solution, PBS), b) anti-mouse CD20 antibody, c) anti-mouse CD20 plus anti-LFA-1/anti-VLA-4 blocking antibodies, d) anti-mouse CD20 combined with bortezomib, or e) anti-mouse CD20 together with bortezomib and anti-LFA-1/anti-VLA-4 antibodies. All drugs were diluted in PBS. Seven days after the start of treatment, the mice were sacrificed by cervical dislocation, and their bone marrow and spleens were harvested for flow cytometric and ELISPOT analysis.

#### Short-term B- and plasma cell-depletion followed by continuous BCD therapy (STD+BCD)

Sixteen-week-old mice (at the age of the clinical onset of disease) received either a) no treatment, b) anti-mouse CD20 plus bortezomib, or c) BCD therapy with anti-mouse CD20 (5 times every 10 days) or, d) treatment b followed by continuous BCD therapy with anti-mouse CD20 (4 times every 10 days after initial treatment).

### Flow cytometric analysis

Fluorescence-activated cell sorting (FACS) staining was performed as described previously [5, 11]. For flow cytometric analysis of plasma cells, after surface staining with anti-CD138 (clone 2–218, BD Pharmingen), we performed intracellular staining for immunoglobulin (Ig)-light chain κ (clone 187.1, DRFZ) and intranuclear BrdU (clone 3D4, BD Pharmingen) staining using the BrdU-Flow-Kit (BD Biosciences) according to the manufacturer’s instructions. To analyze plasma cell subsets, we stained intracellular polyclonal IgG (Southern Biotech) and IgM (clone RMM-1, Biolegend). The antibodies used for B cell staining were anti-CD24 (clone M1/69) and anti-CD117 (clone 2B8) mAbs from BD Pharmingen, anti-IgM (clone RMM-1), anti-CD19 (clone 6D5), anti-CD93 (clone AA4.1) and anti-CD21 (clone 7E9) from Biolegend, and anti-IgD (clone 11.26c), anti-CD23 (clone B3/B4), anti-B220 (clone RA3.6B2) and GL-7 antibodies from DRFZ. The antibodies for the analysis of T cell subsets included anti-CD4 (clone RM4-5), anti-CD8 (clone 53–6.7), anti-CD62L (Clone MEL-14) and anti-CD69 (Clone H1.2F3) all from e-Bioscience, anti-CD3 (Clone 145-2C11, Biolegend), anti-Ly6C (Clone AL-21, BD Pharmingen), and anti-CD44 (clone IM7, DRFZ). Cells were acquired using a FACS BD Canto flow cytometer (Becton-Dickinson) and analyzed using FlowJo software (TreeStar). Absolute cell numbers were calculated based on population frequencies and total cell numbers per organ [11, 15, 16]. The percent remaining cells was then determined by dividing the absolute number of cells in each treated mouse by the mean count obtained in the PBS-treated (control) group and multiplying by 100.

### Detection of antibody-secreting cells by enzyme-linked immunospot assay (ELISPOT)

For detection of anti-double-stranded DNA (dsDNA) antibody-secreting-cells (ASCs), 96-well microtiter plates (Millipore) were pre-coated with methyl-BSA (Sigma Aldrich) and subsequently coated with calf thymus DNA (Sigma Aldrich), as previously described [5, 11, 17]. The spots were developed with 5-bromo-4-chloro-3-indolyphosphate (NBT/BCIP, Thermo Scientific) and enumerated using an automated ELISPOT reader and software (AID Diagnostika).

### Detection of serum antibodies by enzyme-linked immunosorbent assay (ELISA) and detection of proteinuria

Serum was collected from treated and untreated mice at different time points, and IgM- and IgG- anti-dsDNA antibodies were measured by ELISA as described previously using biotin-labeled (detection) goat anti-mouse IgG (γ chain specific) and IgM (μ chain specific) antibodies (Southern Biotech) [6]. Proteinuria was monitored monthly using Albustix (Bayer).

### Statistical analysis

Survival of the mice was analyzed by Kaplan-Meier curves and the effect of treatment on survival by a Cox proportional hazard model. Pairwise comparisons between controls and different treatments were done using post-hoc tests after fitting linear mixed models to avoid the type one error accumulation of single private statistical tests. Statistical analysis was performed with STATA 12 (StataCorp. 2011. Stata Statistical Software: Release 12. College Station, TX: StataCorp LP) and figures were created using GraphPad Prism 5.0 (GraphPad, La Jolla, California, USA). All data were expressed as mean ± SEM.

## Results

### Short-term treatment regimens containing bortezomib lead to effective depletion of plasma cells, including the long-lived compartment

**Fig 1:**
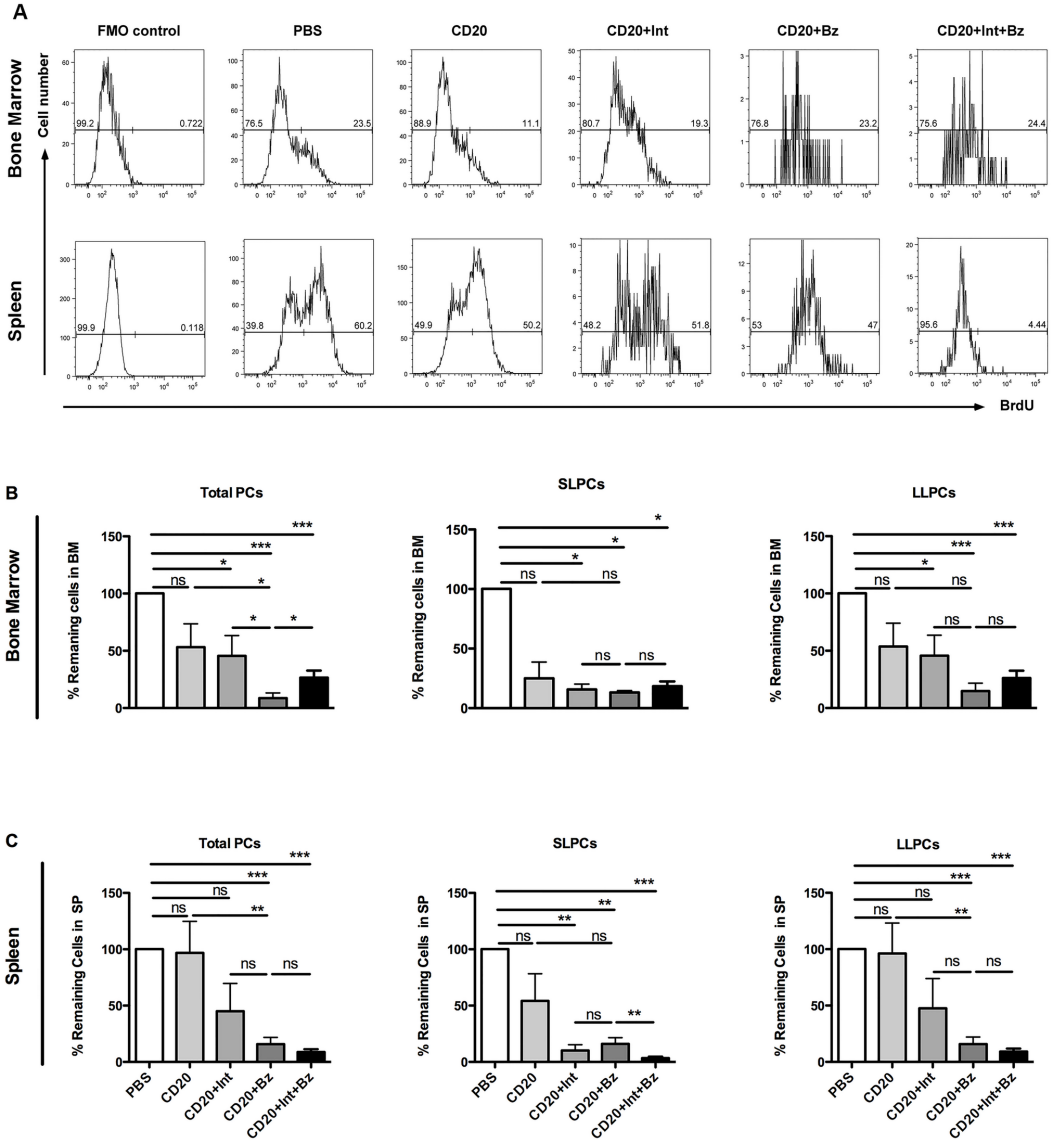
Effects of short-term depletion treatments on plasma cell numbers in bone marrow and spleen. **(A)** Representative FACS histogram of bone marrow and splenic CD138^+^ intracellular κ^+^ BrdU^+^ short-lived plasma cells (SLPCs), and CD138^+^ intracellular κ^+^ BrdU^-^ long-lived plasma cells (LLPCs) from each treatment group. Percentage of remaining cell numbers relative to the control mean of (**B)** bone marrow and (**C)** splenic CD138^+^ intracellular κ^+^ total plasma cells (PCs), SLPCs, and LLPCs in mice treated with PBS, anti-CD20, anti-CD20 plus integrin-blocking antibodies (Int; anti-LFA1 and anti-VLA4 antibodies), anti-CD20 plus bortezomib (Bz) and anti-CD20 plus Int and Bz. Total PCs, SLPCs and LLPCs were enumerated by flow cytometry 7 days after the start of treatment (*n* = 5–6 mice per each group). Values are mean±SEM; ns, non-significant; P>0.05, *P<0.05, **P<0.01, ***P<0.001, post-hoc test. Abbreviations: Bz, bortezomib; CD20, anti-mouse CD20 antibody; FMO, Fluorescence-minus-one; Int, Integrin blocking antibodies; anti-LFA1 and anti-VLA4 antibodies.

Twenty- to 22-week-old female NZB/W F1 mice were treated with a) PBS, b) anti-mouse CD20, c) anti-mouse CD20 plus anti-LFA-1/anti-VLA-4 blocking antibodies, d) anti-mouse CD20 combined with bortezomib, or e) anti-mouse CD20 together with bortezomib and with anti-LFA-1/anti-VLA-4 antibodies. Seven days after treatment, total plasma cells, SLPC and LLPCs in the bone marrow and spleen were enumerated by flow cytometry (Fig 1A). In the bone marrow, total plasma cells (CD138+, intracellular κ^+^) were significantly depleted by the treatments containing plasma cell-targeting agents (anti-CD20/LFA1/VLA4, anti-CD20/Bz and anti-CD20 plus anti-LFA1/anti-VLA4/Bz) to the average of 45%, 9% and 27% respectively, of their original value. In the group receiving anti-CD20 alone, no significant difference in the numbers of total plasma cells was observed (Fig 1B). Of note, combination therapy with anti-CD20, bortezomib and anti-LFA1/anti-VLA4 did not deplete plasma cells better than anti-CD20 plus bortezomib. Bone marrow SLPCs (CD138+, intracellular κ^+^, BrdU^+^) and LLPCs (CD138+, intracellular κ^+^, BrdU^-^) were depleted significantly in all groups treated with plasma cell-targeting agents. Interestingly, more significant reduction of bone marrow LLPCs was achieved by the two bortezomib-based regimens (anti-CD20/Bz and anti-CD20/LFA1/VLA4/Bz), which decreased the average percentage of remaining cells to 15% and 26% respectively (Fig 1B). The effects of the different treatments in the spleen were comparable to those observed in the bone marrow (Fig 1C). Anti-CD20 plus anti-LFA1/anti-VLA4, anti-CD20 plus Bz and anti-CD20 plus anti-LFA1/anti-VLA4/Bz decreased splenic SLPC numbers significantly, to an average of 10%, 16% and 3% remaining cells, respectively (Fig 1C). Conversely, only the bortezomib-based regimens (anti-CD20 plus Bz and anti-CD20 plus anti-LFA1/anti-VLA4 and Bz) achieved significant depletion of splenic LLPCs, which were reduced to an average 16% and 9% respectively, of the original numbers (Fig 1C). Notably, combination therapy with anti-CD20, bortezomib and anti-LFA1/anti-VLA4 led to greater depletion of plasma cells, especially SLPCs in spleen. Anti-CD20 therapy alone showed a trend to deplete only bone marrow and splenic SLPCs, probably by blocking the new generation of SLPCs from B cells. Anti-LFA1/anti-VLA4 plus anti-CD20 antibodies decreased the number of bone marrow and splenic SLPCs significantly, but did not affect LLPCs in spleen. Of all the regimens tested, anti-CD20 in combination with bortezomib was the most effective in depleting both short- and long-lived plasma cells as compared to the other regimens tested.

To determine whether plasma cells secreting antibodies of different isotypes show differences in their susceptibility to the depletion therapies, we enumerated IgM and IgG SLPCs and LLPCs by flow cytometry (S1 Table). In the bone marrow, anti-CD20 treatment significantly depleted all SLPC subpopulations but did not affect LLPCs, supporting the idea that B-cell depletion can reduce the number of newly incoming plasma cells without depleting LLPCs localized in their niche. Anti-CD20 did not deplete any of the plasma cell subpopulations in the spleen (IgM, IgG SLPCs and LLPCs). Importantly, anti-CD20 combined with bortezomib eliminated SLPCs and LLPCs of both isotypes tested (IgM+, IgG+). The addition of anti-LFA1/anti-VLA4 antibodies to the anti-CD20/Bz regimen led to a further significant reduction of only IgM SLPCs in the spleen (S1 Table). In summary, B-cell depletion therapy effectively depleted SLPCs, while the plasma cell-targeting regimens were capable of eliminating IgM and IgG LLPCs. Bortezomib plus anti-CD20 promoted the stronger reduction of bone marrow and splenic IgG-LLPCs.

### Anti-CD20 plus bortezomib efficiently eliminates IgM and IgG anti-dsDNA antibody secreting plasma cells from bone marrow and spleen

**Fig 2:**
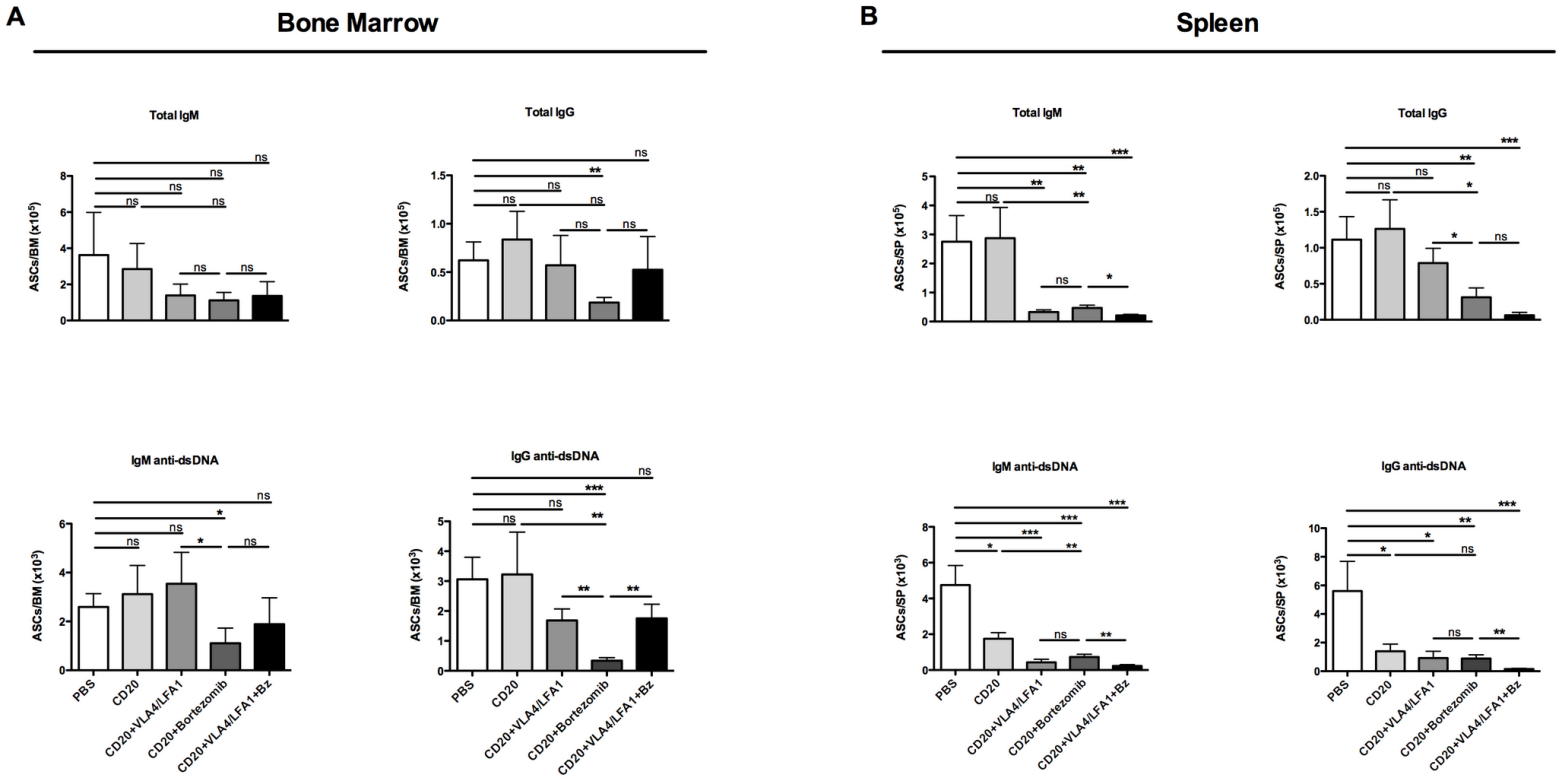
Effects of short-term depletion treatments on the numbers of anti-dsDNA antibody secreting plasma cells in bone marrow and spleen. Absolute number of IgM and IgG together with autoreactive IgM and IgG anti-dsDNA antibody secreting-cells (ASCs) in (**A)** the bone marrow and (**B)** spleen after one-week of treatment in ratio to mean of control, as enumerated by ELISPOT. Values are mean±SEM; ns, non-significant, P>0.05, *P<0.05; **P<0.01, ***P<0.001, by post-hoc test (*n* = 5–6 mice per group). Abbreviations: Bz, bortezomib; CD20, anti-mouse CD20 antibody; Int, integrin-blocking antibodies; anti-LFA1 and anti-VLA4 antibodies.

The efficiency of the different treatments regarding their capacity to eliminate IgM and IgG ASCs and autoreactive plasma cells that secrete anti-dsDNA antibodies was analyzed by ELISPOT. In the bone marrow, IgM ASCs showed resistance to depletion with only a trend towards a decrease when plasma cell-depleting agents were applied. IgG ASCs were significantly depleted only by bortezomib plus anti-CD20. Similarly, only anti-CD20 in combination with bortezomib significantly depleted IgM and IgG anti-dsDNA-secreting cells to the average of 43% and 10% of baseline, respectively (Fig 2A). In the spleen, those regimens containing agents targeting plasma cells (anti-CD20 plus anti-LFA1/anti-VLA4, anti-CD20 plus Bz and anti-CD20 plus anti-LFA1/anti-VLA4/Bz) achieved significant depletion of IgM ASCs but only the bortezomib-based treatment affected IgG ASCs. Autoreactive plasma cells (IgM and IgG anti-dsDNA ASCs) were depleted by all the plasma cell-targeting regimens (Fig 2B). Notably, anti-CD20 alone had no significant effect on IgM and IgG anti-dsDNA antibody-secreting plasma cells in the bone marrow, but induced a significant reduction of both IgM and IgG anti-dsDNA-secreting plasma cells in the spleen (Fig 2A and 2B).

In summary, the fact that BCD did not deplete ASCs (neither IgG nor IgM) or anti-dsDNA-ASCs in the bone marrow confirms that BCD has limited direct effects on bone marrow LLPCs. Anti-CD20 and integrin blocking were only effective in the spleen, where higher numbers of SLPCs are located. This suggests that autoreactive plasma cells in the bone marrow can only be targeted by the proteasome inhibitor bortezomib.

### Impact of different short-term depletive therapies on bone marrow and splenic B cells

In order to further characterize the effects of the treatments on the B-cell populations and their possible role in blocking the pathogenic regeneration plasmablasts/SLPCs and LLPCs, we enumerated different B-cell subsets in bone marrow and spleen by flow cytometry after treatment.

**Fig 3:**
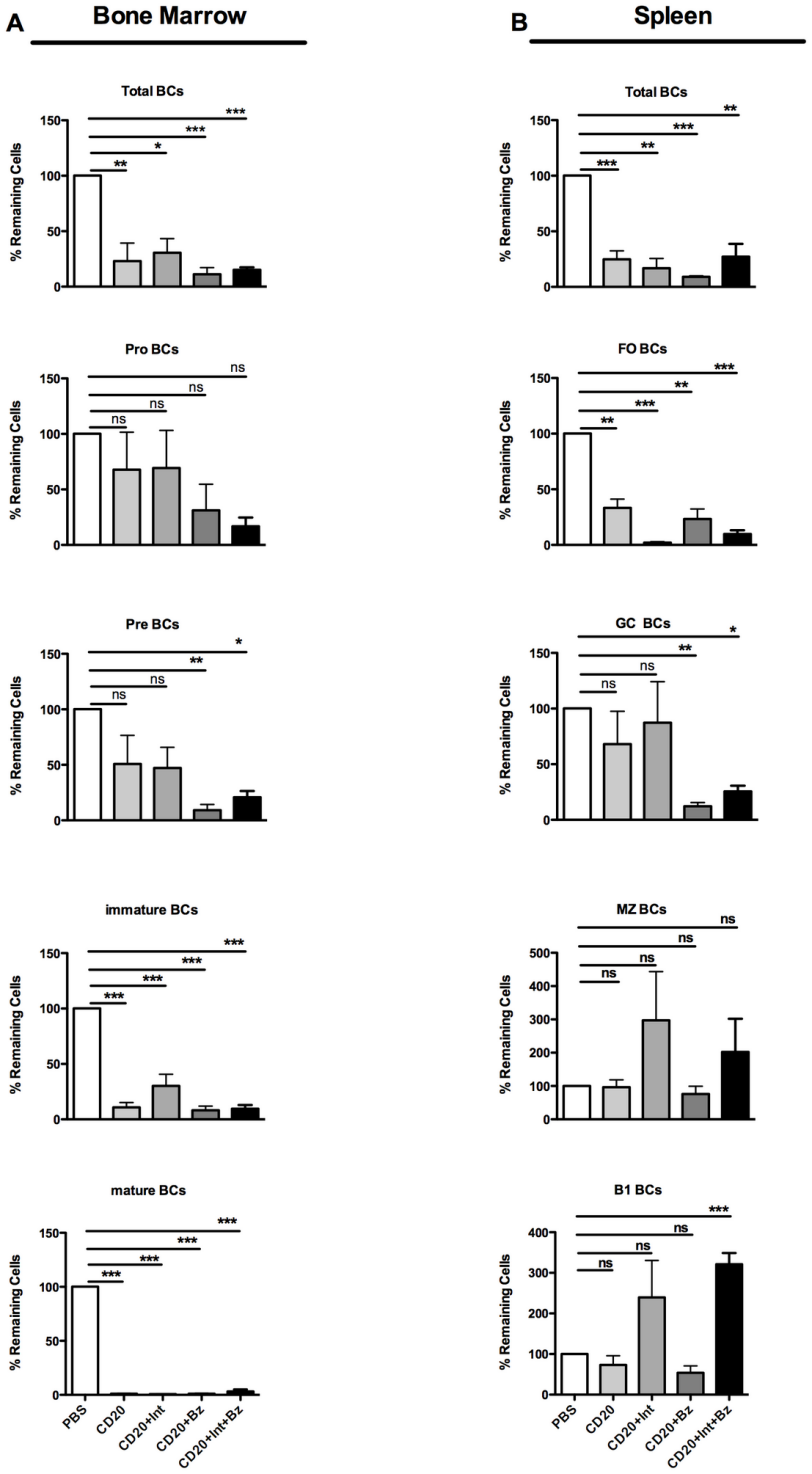
Effects of short-term depletion treatments on the numbers of different B-cell subsets in bone marrow and spleen. Percentage of remaining B cell subsets in the bone marrow and spleen in ratio to the mean of control. (**A)** Bone marrow B-cell subsets identified by flow cytometry: total B cells (BCs) (CD19^+^), bone marrow pro-B cells (CD93^+^CD117^+^), pre-B cells (CD24^+^IgM^-^IgD^-^), immature B cells (CD24^+^IgM^+^IgD^-^), and mature B cells (CD24^-^IgM^+^IgD^+^). (**B)** Splenic B-cell subsets identified by flow cytometry: follicular (FO) B cells (CD23^+^CD21^+^IgM^+^), marginal zone (MZ) B cells (CD23^-^ CD21^+^IgM^+^), germinal center (GC) B cells (IgD^-^GL7^+^), and B1 B cells (CD23^-^CD21^-^IgM^+^). Values are mean±SEM; ns, non-significant, P>0.05, *P<0.05; **P<0.01, ***P<0.001, post-hoc test (*n* = 5–6 mice per group). Abbreviations: Bz, bortezomib; CD20, anti-mouse CD20 antibody; Int, Integrin blocking antibodies; anti-LFA1 and anti-VLA4 antibodies.

Total CD19+ B cells in the bone marrow and spleen were depleted significantly by all treatments; anti-CD20 plus bortezomib achieved the strongest effect (Fig 3A and 3B). Anti-CD20 alone did not significantly affect early-stage B cells (pro- and pre-B cells) in the bone marrow, but depleted immature and mature B cells significantly (Fig 3A). Anti-CD20 plus anti-LFA1/anti-VLA4 achieved similar results. In contrast, the bortezomib-based treatments (anti-CD20 plus bortezomib and anti-CD20 plus anti-LFA1/anti-VLA4/Bz) substantially reduced pro-B cells and significantly decreased pre-, immature and mature B cells (Fig 3A). In the spleen, all treatments significantly reduced the number of follicular (FO) B cells, but anti-CD20 plus anti-LFA1/anti-VLA4 and anti-CD20 plus anti-LFA1/anti-VLA4/Bz achieved the greatest reductions (Fig 3B). Germinal center (GC) B cells were significantly decreased by anti-CD20 plus bortezomib and anti-CD20 plus anti-LFA1/anti-VLA4/Bz. Marginal zone (MZ) B cells and B1 B cells were resistant to all treatments. MZ and B1 B cell numbers were actually higher in the groups treated with anti-LFA1/anti-VLA4 antibodies (Fig 3B). These data show that anti-CD20 antibody can deplete immature and mature B cells in the spleen and bone marrow without affecting early-stage (pro- and pre-B cells), MZ and B1 B cells. The addition of anti-LFA1/anti-VLA4 antibodies to the regimen did not increase the BCD effect, but increased the number of MZ and B1 B cells. Interestingly, the bortezomib-based treatments achieved efficient depletion of pre-B cells and germinal center B cells. The reduction of these cells can be considered a direct effect of bortezomib on cells with high protein synthesis and proliferation rates, as previously shown for germinal center B cells in lupus mice [10].

**Fig 4:**
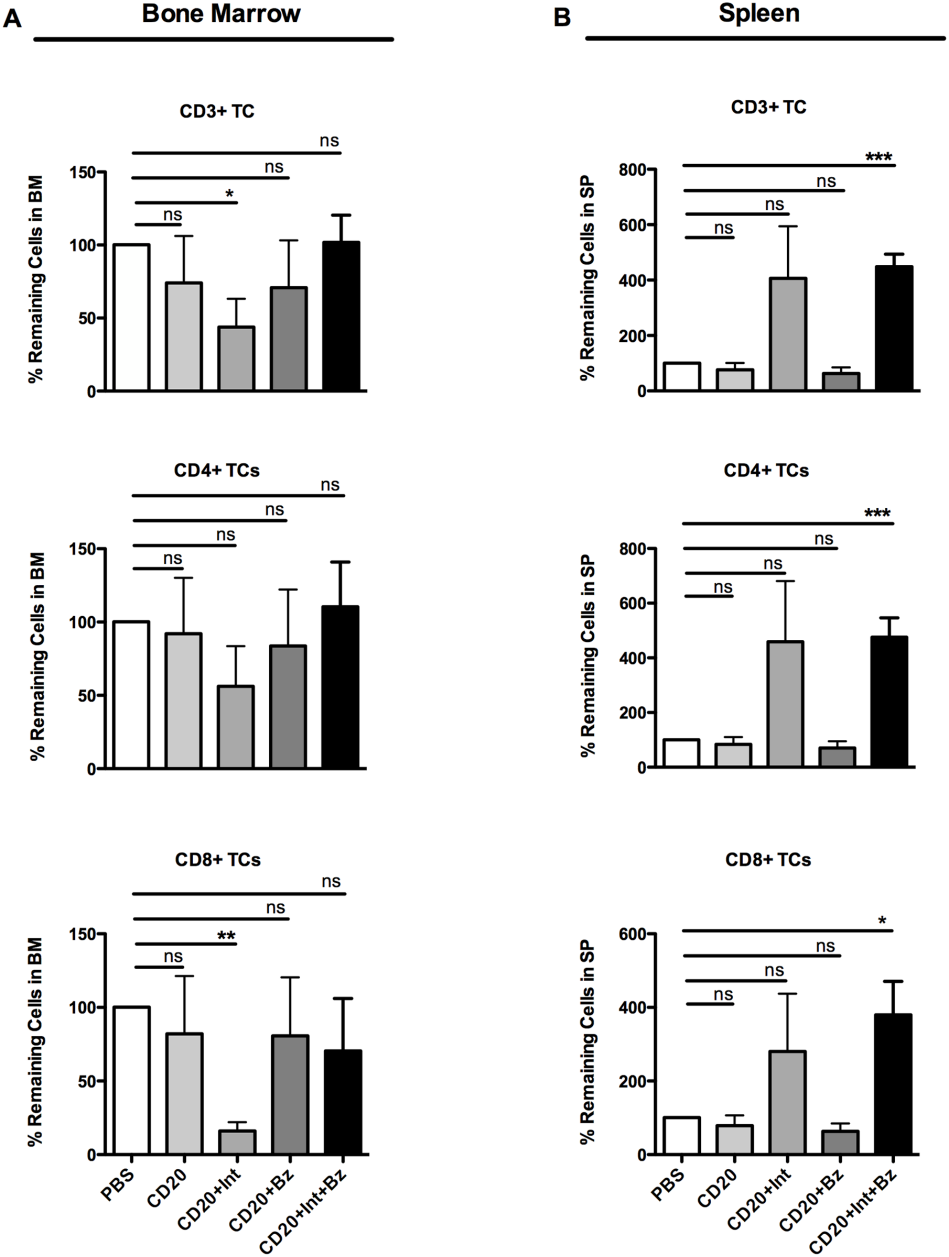
Effects of short-term depletion treatments on bone marrow and splenic T cells. Percentage of remaining CD3^+^ T cells, CD4^+^ T-helper cells, and CD8^+^ T-cytotoxic cells after one week of treatment in ratio to the mean of control in (**A)** the bone marrow, and (**B)** spleen. Values are mean±SEM; ns, non-significant, P>0.05, *P<0.05, **P<0.01, ***P<0.001, post-hoc test (*n* = 5–6 mice per group). Abbreviations: Bz, bortezomib; CD20, anti-mouse CD20 antibody; Int, Integrin blocking antibodies, anti-LFA1 and anti-VLA4 antibodies.

We also characterized the effects of the regimens on T cells. In the bone marrow, total CD3+, CD4+ and CD8+ T cell subsets were not influenced by the B-cell depletion alone or in combination with the bortezomib-based regimens (Fig 4A). Anti-CD20 plus anti-LFA1/anti-VLA4 led to a slight and significant reduction of bone marrow CD4+ and CD8+ T cells, respectively. In the spleen, total CD3+, CD4+ and CD8+ T-cell subsets did not change after treatment with anti-CD20 with or without bortezomib (Fig 4B). However, in the groups treated with anti-integrin antibodies, an increase in all splenic T-cell subsets was observed. These data show that anti-LFA1/anti-VLA4 treatment induces an increase in T cells in the spleen and a concomitant reduction in the bone marrow. Conversely anti-CD20 plus bortezomib treatment did not have any effect on T-cell subsets.

### Plasma cell depletion using bortezomib followed by continuous BCD therapy delays the onset of disease

The data presented above shows that bortezomib in combination with anti-CD20 antibody was most effective in depleting LLPCs, and achieved a significant depletion of the B cells (mature and GC B cells) that may be responsible for the described plasma cell re-generation [18]. We have previously shown that the sustained therapeutic elimination of autoreactive LLPCs requires both the depletion of these cells and the inhibition of their regeneration by means of a maintenance therapy that depletes LLPC precursors and prevents their differentiation into LLPCs. [11]. Therefore, the combination of plasma cell ablation with the efficient and preferably selective ablation of the autoreactive LLPC precursors could represent a new and useful strategy in antibody-mediated autoimmune diseases. To further investigate this hypothesis and to analyze whether this depletive treatment influences the onset and the progression of disease, we treated NZB/W F1 mice as follows: a) untreated control group, b) short-term depletion (STD) of B and plasma cells with anti-CD20 plus bortezomib, c) B-cell depletion (BCD) (5 times every 10 days), and d) treatment as group b followed by continuous BCD with anti-CD20 antibody (4 times every 10 days). The following outcome variables were monitored: serum autoantibody levels, onset and extent of proteinuria, and survival rate.

**Fig 5:**
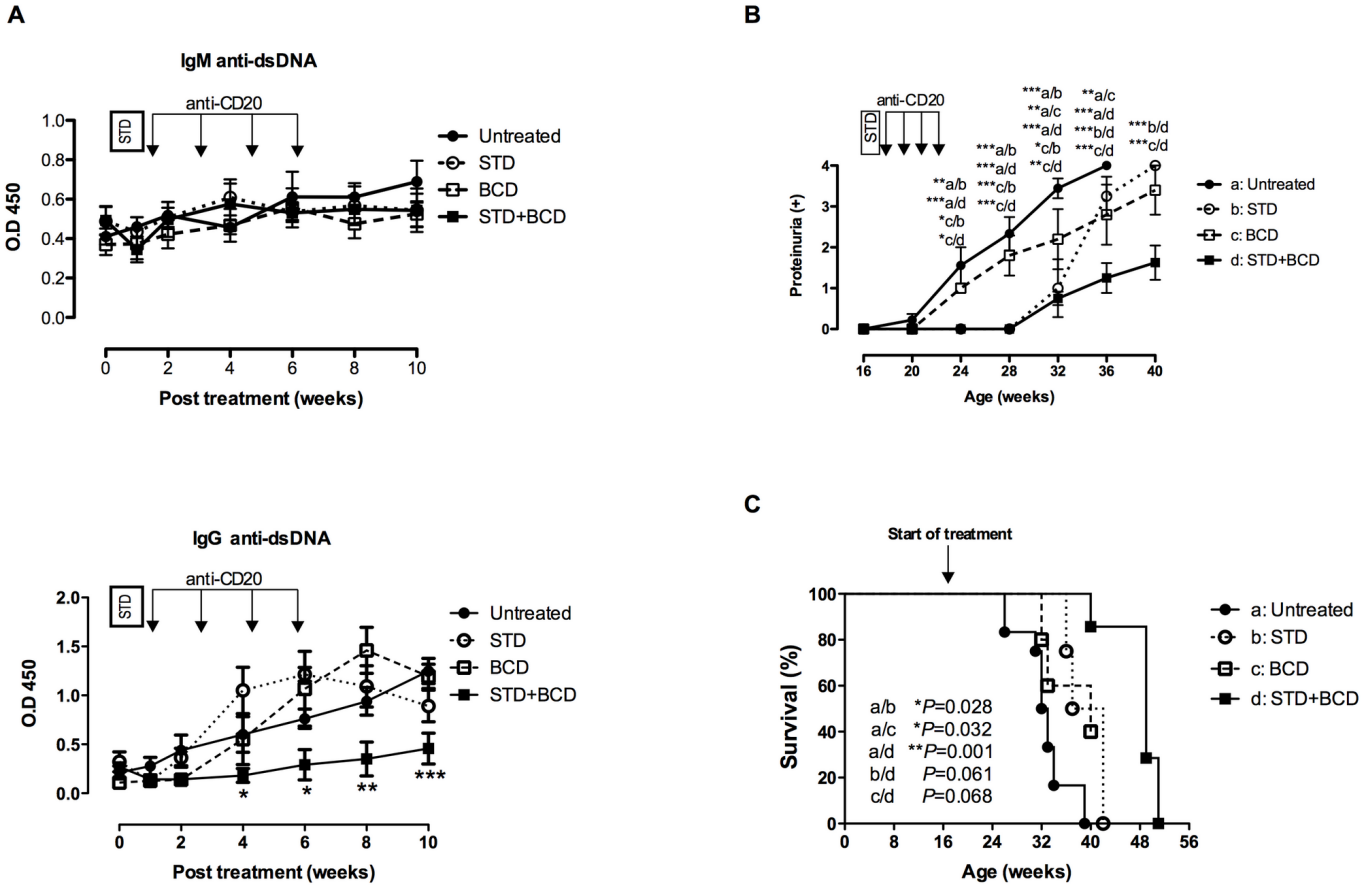
B cell depletion (BCD) maintenance therapy after short-term depletion (STD) of B and plasma cells with ant-CD20 and bortezomib improves the disease in NZB/W F1 mice. Mice (n = 4) were treated with anti-CD20 and bortezomib (STD) alone or continuous B cell depletion without bortezomib (BCD, n = 5) or treated as STD followed by BCD maintenance therapy with anti-CD20 (STD+BCD, n = 4). (**A)** Serum IgM and IgG anti-dsDNA antibody levels in treated and untreated mice (n = 9), as measured by ELISA. (**B)** Proteinuria in treated and untreated mice. Statistical differences between treated and untreated mice were analyzed using the post-hoc test (ns, non-significant, P>0.05, *P<0.05; **P<0.01, ***P<0.001). (**C)** Survival curves for treated and untreated NZB/W F1 mice (Kaplan—Meier log-rank test). Abbreviations: STD, Short-term depletion (anti-CD20 and bortezomib); BCD; B cell depletion (anti-CD20).

In all treatment groups, IgM anti-dsDNA antibody levels declined the first week after treatment and reached the levels observed in untreated mice one week later (Fig 5A). After depletion therapy, IgG anti-dsDNA antibody levels declined in all treated groups, but remained significantly lower only in the initial STD plus continuous BCD therapy group (Fig 5A).

Compared to untreated mice (group a), those receiving STD plus continuous BCD therapy (group d) showed a significant delay in the onset of proteinuria from age 24 to 32 weeks (Fig 5B). Notably, the group treated with STD alone (group b), also showed a delay in the onset of proteinuria (from week 24 to week 32), suggesting that a single cycle of bortezomib and anti-CD20 could have long-lasting beneficial effects in NZB/WF1 mice. BCD therapy without bortezomib (group c) did not delay the onset of proteinuria but induced a slight reduction of the proteinuria levels, which became significant at weeks 32 and 36. However, only the combination of STD with continuous BCD using anti-CD20 antibody promoted a significant delay in the onset of proteinuria followed by a persistent decrease in proteinuria levels from week 36 to the end of the observation period (week 40) as compared to untreated mice, and mice treated with BCD or STD alone over time (*P*<0.001, *P* = 0.003, *P* = 0.067, respectively) (Fig 5B). In line with the delayed onset and development of proteinuria, the mice treated with STD alone (group b), BCD (group c), and STD in combination with continuous BCD therapy (group d) survived longer than untreated mice (*P* = 0.028 and *P* = 0.032, *P* = 0.001, respectively). Likewise, mice treated with initial STD plus continuous BCD therapy had higher survival rates than those treated with STD and BCD alone (*P* = 0.061, *P* = 0.068) (Fig 5C).

These data show that continuous BCD therapy after efficient B cell and plasma cell depletion reduces the autoantibody levels and ameliorates nephritis, promoting the survival of lupus-prone mice.

## Discussion and Conclusions

Autoantibodies play a crucial role in the pathogenesis of many autoimmune diseases. Therefore, their reduction or removal is an important therapeutic goal. Previously, we showed that autoantibodies can be generated by two different plasma cell compartments. The first consists of short-lived plasmablasts and plasma cells recently generated from activated B cells [4]. Therapies targeting B cells block the generation of these newly generated autoantibody-secreting plasmablasts, and immunosuppressive drugs that affect proliferating B cells and plasmablasts can also eliminate this compartment [8]. The second compartment consists of long-lived (memory) plasma cells that reside in survival niches in the bone marrow and in inflamed tissues [8, 19]. In contrast to the first compartment, the second is resistant to B-cell targeting and conventional immunosuppressive therapies [5, 7, 8]. Compelling evidence suggests that these autoreactive LLPCs can drive autoantibody-mediated inflammation, the maintenance of autoimmunity and refractory autoimmune disease [8]. Therefore, there is an urgent need to define more effective ways to eliminate the long-lived plasma cell compartment in autoimmune diseases.

In this study, we explored the short-term effects of different depletion strategies, targeting B cells alone or in combination with plasma cells in NZB/W F1 mice, a model of anti-dsDNA antibody-driven nephritis that resembles lupus nephritis. We showed that B-cell depletion alone did not significantly reduce the number of short- and long-lived plasma cells in the bone marrow and spleen. We observed a reduction of bone marrow IgG and IgM SLPCs and of splenic autoreactive plasma cells after BCD. This observation supports the idea that BCD does not affect the existing LLPCs pool, but could partially slow down the ingress of SLPCs (including the autoreactive ones) into the bone marrow. However, this treatment promoted only a partial reduction of the number of splenic SLPCs and IgM ASCs suggesting that the a single cycle of anti-CD20 may not be effective for significantly reducing the constant generation of autoreactive plasma cells from BCD-resistant precursors (i.e., germinal center, marginal zone and B1 B cells). A significant reduction of BrdU-positive short-lived bone marrow and splenic plasma cells was only observed for BCD combined with plasma cell depletion (anti-LFA1/anti-VLA4 and/or Bz). As expected, the LLPC compartments (particularly bone marrow IgG ASCs, including anti-dsDNA-IgG autoreactive plasma cells) were resistant to BCD alone and could be targeted only by plasma cell-depleting treatments (i.e., anti-LFA1/VLA4 and bortezomib). This confirms data from murine models [9, 20, 21] and humans demonstrating the maintenance of total IgG, including antimicrobial antibodies, after BCD [22, 23] and the heterogeneous response to BCD in autoimmunity [8]. It also underlines the independence of LLPC and B-cell compartments. Notably, we show that blockade of LFA-1 and VLA-4, described as LLPC-depleting treatment in non-autoimmune mice [9], was not as efficient as bortezomib-based treatments in depleting LLPCs and autoreactive bone marrow plasma cells in our autoimmune model. We also observed a different response of bone marrow and spleen ASCs to integrin blocking: the latter were further depleted after addition of integrin blocking to the bortezomib and anti-CD20 combination. This suggests that bone marrow LLPCs in NZB/W F1 lupus mice could be less dependent on signals provided by LFA-1 and VLA-4, and that other signals provided by the survival niche in these mice may overcome the blocking of this pathway (e.g., higher APRIL and BAFF levels [24] or higher CXCL12 expression [25].

Our data indicate that effective depletion of long-lived and autoreactive plasma cells can only be achieved by depletion regimens containing the proteasome inhibitor bortezomib. The combination of bortezomib with integrin blockade does not seem to provide an additional synergistic effect. Beyond their role in promoting the interaction between stromal cells and plasma cells in the bone marrow niches, LFA-1 and VLA-4 adhesion molecules are predominantly involved in leukocyte trafficking and extravasation [26]. In line with this, our data shows that anti-LFA1/anti-VLA4 blocking antibodies increase the number of T cells, marginal zone and B1 B cells in the spleen of treated mice. Considering all these observations, and the potential role of marginal zone and B1 B cells in the pathogenesis of SLE [27], the use of anti-LFA1/anti-VLA4 antibodies alone does not appear to be a good option for the depletion of long-lived plasma cells in autoimmunity. However, the interesting hypothesis that this treatment can be used after LLPC mobilization to avoid the re-entry of these cells in their niches deserves further investigation.

We have previously shown that long-term targeting of plasma cell precursors with cyclophosphamide in combination with bortezomib for short-term depletion of plasma cells, including LLPCs is able to reduce the regeneration of the plasma cell compartment after plasma cell depletion [11]. In the current study, B cells were selectively depleted by a monoclonal anti-mouse CD20 antibody. This combination treatment has the evident advantage of immediately ablating of LLPCs and the B cells that sustain their regeneration (i.e., germinal center and follicular B cells) without affecting T cells. The addition of bortezomib to BCD may indeed promote a stronger depletion of the cells that fuel the autoreactive loop and are resistant to BCD (e.g. germinal center B cells). Therefore, this combination therapy can promote immediate LLPC depletion and at the same time may effectively reduce the ongoing generation of plasma cells. In line with this hypothesis, the autoreactive antibody titers could be kept persistently low by administrating BCD maintenance therapy after short-term depletion with anti-CD20 plus bortezomib. This is likely due to the capacity of this combination therapy to block the plasma cell regeneration, as was recently suggested [11]. Wang et al. [28] describe a reduction of plasma cells in the spleen, bone marrow and inflamed kidneys after long-term treatment (12 weeks) with the same monoclonal anti-CD20 antibody that was used in this study. Unfortunately, the question of whether this long-term BCD regimen really affected the long-lived memory plasma cell compartment remains unclear since the authors did not distinguish between short-lived and long-lived plasma cells. However, the authors describe a significant reduction of the number of IgG- and dsDNA-specific antibody-secreting cells in the spleen with only a modest decrease in IgG- and dsDNA-specific plasma cells in the bone marrow. As also discussed by the authors, this is likely due to resistance of bone marrow LLPCs to long-term BCD and to the consistent reduction only those SLPCs that are directly eliminated by BCD and the consequent interruption of ongoing plasma cell generation [28]. In agreement with data reported in this and our previous study [11], this finding suggests that long-term anti-CD20 therapy may indeed reduce the production of autoreactive SLPCs. However, the therapeutic depletion of autoreactive memory LLPCs would require the direct targeting of these cells. Accordingly, BCD showed no effect on anti-dsDNA antibody levels in NZB/W F1 mice treated once a week for 4 weeks with anti-CD20 [29]. This supports the idea that the elimination of the preexisting plasma cell compartment, especially memory LLPCs, is required to induce an immediate decrease in autoreactive antibody titers. Therefore, the combination of bortezomib plus anti-CD20 could overcome the limitations of B-cell depletion alone, which has no effect on LLPCs, while at the same time reducing the need for chronic bortezomib treatment, which has been associated with neurological and hematological side effects [30]. In line with this, we show here that a short-term plasma cell depletion regimen including bortezomib quickly reduces IgG anti-dsDNA antibody levels, resulting in a significant delay in the onset of proteinuria. However, due to B cell hyperactivity, IgG anti-dsDNA autoantibodies levels increased after short-term treatment and, at the end of the observation period, all mice showed autoantibody titers similar to those in untreated controls. A key finding was that the administration of four cycles of BCD after plasma cell depletion with bortezomib kept IgG anti-dsDNA autoantibody levels at lower levels with an effect lasting four additional weeks after the final cycle. Another noteworthy finding was that IgM anti-dsDNA antibodies declined only transiently in response to both short-term depletion and continuous anti-CD20 treatment. This may be due to resistance to BCD and bortezomib by innate B cells (B1 and marginal zone B cells) that form the main source of IgM antibodies, as described above. Also, the fact that the reduction of IgG anti-dsDNA antibodies, which play major role in the development of nephritis in NZB/W F1 mice [31], was related to a significant delay in nephritis and to an increase the survival rate in NZB/W F1 mice supports the clinical effectiveness of this treatment approach. Our data show a significant improvement of disease manifestations and a delay in the onset of proteinuria (from week 24 to week 32) and prolonged survival in both the STD group treated with bortezomib plus CD20 and in the mice treated with STD plus continuous BCD therapy. Therefore, a single cycle of bortezomib and anti-CD20 could have a short-term beneficial effect in NZB/W F1 mice. Confirming previous finding [29], a noteworthy finding here was that BCD alone was not able to decrease the levels of serum auto-antibodies, but could reduce the progression of nephritis. This effect was indeed long-lasting and associated with an increase in the survival rate. However, proteinuria was progressive and a significant reduction was visible only from 32 weeks of age. Extending prior observations [11, 29], we showed that the elimination of the LLPCs by combining of the STD therapy with continuous anti-CD20 therapy lowered IgG anti-dsDNA antibody levels and promoted a persistent decrease in the level of proteinuria levels (by an average ≤200 mg/dL) from week 24 to the end of the observation period (week 40) as compared to untreated mice and mice treated with STD alone; consequently, this increased the survival of the mice to 50 weeks of age. Therefore, the clinical effect of the combination therapy is significantly higher than that of STD or BCD alone. This confirms the hypothesis that this treatment option can combine two positive effects: effective elimination of the autoreactive IgG-LLPCs secreting the autoantibodies responsible for disease chronicity and interruption of the generation of new autoreactive plasmablasts and SLPCs. We speculate that a longer continuation of the BCD therapy would keep the anti-dsDNA antibody levels low and prevent development of nephritis as long as the treatment will be performed, which we plan to do in a bigger study. One limitation of this study is the lack of an extensive characterization of the kinetics of plasma cells and autoreactive ASCs depletion and re-generation over the long term. However, our previous study [11] demonstrated that a cycle of bortezomib combined with maintenance therapy for depletion of the precursors of LLPCs could promote the persistent depletion of LLPCs. Here, we show that anti-CD20 depleting antibody promotes a persistent decline in anti-dsDNA serum antibody levels. This finding, together with growing evidences in the literature showing that BCD is effective in reducing the production of SLPCs without affecting the LLPC compartment, [9, 28, 29] strongly supports the hypothesis that this combination therapy regimen is able to promote sustained plasma cell depletion leading to a long-lasting clinical benefit.

Altogether, our results suggest that anti-CD20 plus bortezomib is the best and most efficient method for substantially depleting LLPCs and promoting the quick reduction of IgG-anti-dsDNA antibodies. Moreover, this regimen has the advantage of immediate ablation of the B cells that fuel the autoreactive loop (i.e., germinal center and follicular B cells). This initial depletion could be complemented by a BCD maintenance regimen that efficiently and selectively ablates the precursors of autoreactive LLPCs, thus preventing their re-generation, as was recently proposed both for mice [11] and SLE patients [12].

It is difficult to directly compare the NZB/W F1 mouse model of lupus with the SLE patients due to the use of different antibodies for B-cell depletion and the scarcity of data on the depletion of CD20-expressing B cells in solid tissues of SLE patients. However, as observed in NZB/W F1 mice, there is a subpopulation of SLE patients with high serum levels of pathogenic anti-dsDNA antibodies that are secreted by both short-lived plasmablasts (due to B cell hyperactivity) and long-lived memory plasma cells [12, 32]. A clinical study is needed to determine whether, the combination of plasma cell depletion with bortezomib followed by efficient maintenance therapy that targets B cells is really effective in the treatment in SLE patients.

This study provides important new information on therapeutic B-cell and plasma cell targeting in lupus. Our data suggest that the regimen that best targets plasma cells while quickly and significantly reducing autoantibodies levels contains the proteasome inhibitor bortezomib. In conclusion, this suggests that the elimination of autoreactive long-lived plasma cells and the concomitant prevention of their regeneration could be a promising therapeutic option for SLE and other antibody-mediated diseases.

## Supporting Information

S1 Table Percentage of remaining cell numbers of plasma cell subsets compared to the control group in bone marrow and spleen after one-week treatment. (PDF)

## References

[1] Kotzin BL. Systemic lupus erythematosus. Cell. 1996;85(3):303–6. Epub 1996/05/03. . 8616885 10.1016/s0092-8674(00)81108-3

[2] Sherer Y, Gorstein A, Fritzler MJ, Shoenfeld Y. Autoantibody explosion in systemic lupus erythematosus: more than 100 different antibodies found in SLE patients. Seminars in arthritis and rheumatism. 2004;34(2):501–37. Epub 2004/10/27. . 15505768 10.1016/j.semarthrit.2004.07.002

[3] Manz RA, Thiel A, Radbruch A. Lifetime of plasma cells in the bone marrow. Nature. 1997;388(6638):133–4. Epub 1997/07/10. 10.1038/40540 . 9217150

[4] Slifka MK, Antia R, Whitmire JK, Ahmed R. Humoral immunity due to long-lived plasma cells. Immunity. 1998;8(3):363–72. Epub 1998/04/07. . 9529153 10.1016/s1074-7613(00)80541-5

[5] Hoyer BF, Moser K, Hauser AE, Peddinghaus A, Voigt C, Eilat D, et al Short-lived plasmablasts and long-lived plasma cells contribute to chronic humoral autoimmunity in NZB/W mice. The Journal of experimental medicine. 2004;199(11):1577–84. Epub 2004/06/03. 10.1084/jem.20040168 15173206 PMC2211779

[6] Cheng Q, Mumtaz IM, Khodadadi L, Radbruch A, Hoyer BF, Hiepe F. Autoantibodies from long-lived 'memory' plasma cells of NZB/W mice drive immune complex nephritis. Annals of the rheumatic diseases. 2013;72(12):2011–7. Epub 2013/10/12. 10.1136/annrheumdis-2013-203455 . 24114925

[7] Mumtaz IM, Hoyer BF, Panne D, Moser K, Winter O, Cheng QY, et al Bone marrow of NZB/W mice is the major site for plasma cells resistant to dexamethasone and cyclophosphamide: implications for the treatment of autoimmunity. Journal of autoimmunity. 2012;39(3):180–8. Epub 2012/06/26. 10.1016/j.jaut.2012.05.010 . 22727274

[8] Hiepe F, Dörner T, Hauser AE, Hoyer BF, Mei H, Radbruch A. Long-lived autoreactive plasma cells drive persistent autoimmune inflammation. Nature reviews Rheumatology. 2011;7(3):170–8. Epub 2011/02/02. 10.1038/nrrheum.2011.1 . 21283146

[9] DiLillo DJ, Hamaguchi Y, Ueda Y, Yang K, Uchida J, Haas KM, et al Maintenance of long-lived plasma cells and serological memory despite mature and memory B cell depletion during CD20 immunotherapy in mice. J Immunol. 2008;180(1):361–71. Epub 2007/12/22. . 18097037 10.4049/jimmunol.180.1.361

[10] Neubert K, Meister S, Moser K, Weisel F, Maseda D, Amann K, et al The proteasome inhibitor bortezomib depletes plasma cells and protects mice with lupus-like disease from nephritis. Nature medicine. 2008;14(7):748–55. Epub 2008/06/11. 10.1038/nm1763 . 18542049

[11] Taddeo A, Khodadadi L, Voigt C, Mumtaz IM, Cheng Q, Moser K, et al Long-lived plasma cells are early and constantly generated in New Zealand Black/New Zealand White F1 mice and their therapeutic depletion requires a combined targeting of autoreactive plasma cells and their precursors. Arthritis research & therapy. 2015;17(1):39 Epub 2015/04/19. 10.1186/s13075-015-0551-3 25889236 PMC4411657

[12] Alexander T, Sarfert R, Klotsche J, Kuhl AA, Rubbert-Roth A, Lorenz HM, et al The proteasome inhibitior bortezomib depletes plasma cells and ameliorates clinical manifestations of refractory systemic lupus erythematosus. Annals of the rheumatic diseases. 2015;74(7):1474–8. Epub 2015/02/25. 10.1136/annrheumdis-2014-206016 . 25710470 PMC4484251

[13] Haas KM, Watanabe R, Matsushita T, Nakashima H, Ishiura N, Okochi H, et al Protective and pathogenic roles for B cells during systemic autoimmunity in NZB/W F1 mice. J Immunol. 2010;184(9):4789–800. Epub 2010/04/07. 10.4049/jimmunol.0902391 . 20368280 PMC3734557

[14] Jacobi AM, Mei H, Hoyer BF, Mumtaz IM, Thiele K, Radbruch A, et al HLA-DRhigh/CD27high plasmablasts indicate active disease in patients with systemic lupus erythematosus. Annals of the rheumatic diseases. 2010;69(1):305–8. Epub 2009/02/07. 10.1136/ard.2008.096495 . 19196727

[15] Chervenick PA, Boggs DR, Marsh JC, Cartwright GE, Wintrobe MM. Quantitative studies of blood and bone marrow neutrophils in normal mice. The American journal of physiology. 1968;215(2):353–60. Epub 1968/08/01. . 5665168 10.1152/ajplegacy.1968.215.2.353

[16] Benner R, Hijmans W, Haaijman JJ. The bone marrow: the major source of serum immunoglobulins, but still a neglected site of antibody formation. Clinical and experimental immunology. 1981;46(1):1–8. Epub 1981/10/01. 7039877 PMC1536329

[17] Cassese G, Lindenau S, de Boer B, Arce S, Hauser A, Riemekasten G, et al Inflamed kidneys of NZB / W mice are a major site for the homeostasis of plasma cells. European journal of immunology. 2001;31(9):2726–32. Epub 2001/09/06. . 11536171 10.1002/1521-4141(200109)31:9<2726::aid-immu2726>3.0.co;2-h

[18] Taddeo A, Hoyer BF, Chang HD, Radbruch A, Hiepe F. Targeting autoreactive plasma cells in autoimmunity: a new treatment approach combining plasma cell and B cell depletion. 32nd EWRR; February 23–25 2012; Stockholm: Ann Rheum Dis; 2012.

[19] Radbruch A, Muehlinghaus G, Luger EO, Inamine A, Smith KG, Dorner T, et al Competence and competition: the challenge of becoming a long-lived plasma cell. Nature reviews Immunology. 2006;6(10):741–50. Epub 2006/09/16. 10.1038/nri1886 . 16977339

[20] Huang H, Benoist C, Mathis D. Rituximab specifically depletes short-lived autoreactive plasma cells in a mouse model of inflammatory arthritis. Proceedings of the National Academy of Sciences of the United States of America. 2010;107(10):4658–63. Epub 2010/02/24. 10.1073/pnas.1001074107 20176942 PMC2842072

[21] Ahuja A, Anderson SM, Khalil A, Shlomchik MJ. Maintenance of the plasma cell pool is independent of memory B cells. Proceedings of the National Academy of Sciences of the United States of America. 2008;105(12):4802–7. Epub 2008/03/15. 10.1073/pnas.0800555105 18339801 PMC2290811

[22] Cambridge G, Leandro MJ, Teodorescu M, Manson J, Rahman A, Isenberg DA, et al B cell depletion therapy in systemic lupus erythematosus: effect on autoantibody and antimicrobial antibody profiles. Arthritis and rheumatism. 2006;54(11):3612–22. Epub 2006/11/01. 10.1002/art.22211 . 17075806

[23] Vital EM, Dass S, Buch MH, Henshaw K, Pease CT, Martin MF, et al B cell biomarkers of rituximab responses in systemic lupus erythematosus. Arthritis and rheumatism. 2011;63(10):3038–47. Epub 2011/05/28. 10.1002/art.30466 . 21618204

[24] Vincent FB, Morand EF, Schneider P, Mackay F. The BAFF/APRIL system in SLE pathogenesis. Nature reviews Rheumatology. 2014;10(6):365–73. Epub 2014/03/13. 10.1038/nrrheum.2014.33 . 24614588

[25] Wang A, Guilpain P, Chong BF, Chouzenoux S, Guillevin L, Du Y, et al Dysregulated expression of CXCR4/CXCL12 in subsets of patients with systemic lupus erythematosus. Arthritis and rheumatism. 2010;62(11):3436–46. Epub 2010/08/20. 10.1002/art.27685 . 20722038 PMC8972909

[26] Yusuf-Makagiansar H, Anderson ME, Yakovleva TV, Murray JS, Siahaan TJ. Inhibition of LFA-1/ICAM-1 and VLA-4/VCAM-1 as a therapeutic approach to inflammation and autoimmune diseases. Medicinal research reviews. 2002;22(2):146–67. Epub 2002/02/22. . 11857637 10.1002/med.10001

[27] Anolik J, Sanz I. B cells in human and murine systemic lupus erythematosus. Current opinion in rheumatology. 2004;16(5):505–12. Epub 2004/08/18. . 15314486 10.1097/01.bor.0000133660.52599.f6

[28] Wang W, Rangel-Moreno J, Owen T, Barnard J, Nevarez S, Ichikawa HT, et al Long-term B cell depletion in murine lupus eliminates autoantibody-secreting cells and is associated with alterations in the kidney plasma cell niche. J Immunol. 2014;192(7):3011–20. Epub 2014/02/28. 10.4049/jimmunol.1302003 24574498 PMC3965596

[29] Bekar KW, Owen T, Dunn R, Ichikawa T, Wang W, Wang R, et al Prolonged effects of short-term anti-CD20 B cell depletion therapy in murine systemic lupus erythematosus. Arthritis and rheumatism. 2010;62(8):2443–57. Epub 2010/05/28. 10.1002/art.27515 20506300 PMC2920998

[30] Corso A, Mangiacavalli S, Varettoni M, Pascutto C, Zappasodi P, Lazzarino M. Bortezomib-induced peripheral neuropathy in multiple myeloma: a comparison between previously treated and untreated patients. Leukemia research. 2010;34(4):471–4. Epub 2009/08/14. 10.1016/j.leukres.2009.07.022 . 19674790

[31] Ohnishi K, Ebling FM, Mitchell B, Singh RR, Hahn BH, Tsao BP. Comparison of pathogenic and non-pathogenic murine antibodies to DNA: antigen binding and structural characteristics. International immunology. 1994;6(6):817–30. Epub 1994/06/01. . 8086372 10.1093/intimm/6.6.817

[32] Alexander T, Thiel A, Rosen O, Massenkeil G, Sattler A, Kohler S, et al Depletion of autoreactive immunologic memory followed by autologous hematopoietic stem cell transplantation in patients with refractory SLE induces long-term remission through de novo generation of a juvenile and tolerant immune system. Blood. 2009;113(1):214–23. Epub 2008/10/01. 10.1182/blood-2008-07-168286 . 18824594

