# Supplementary material for: Bortezomib Plus Continuous B Cell Depletion Results in Sustained Plasma Cell Depletion and Amelioration of Lupus Nephritis in NZB/W F1 Mice
Source: PLoS One. 2015 Aug 7;10(8):e0135081. doi: 10.1371/journal.pone.0135081 (PMC4529137; doi:10.1371/journal.pone.0135081)
Supplement: S1 Table — (PDF) [file pone.0135081.s001.pdf]

Table 1. Percentage of remaining cell numbers of plasma cell subsets compared to the control group in bone marrow and spleen after one-week treatment.

Tissue	Treatment	SLPC (%)		LLPC (%)	
		IgM+	IgG+	IgM+	IgG+
**Bone Marrow**					
	CD20	24 ± 14***	17 ± 5*	48 ± 18	42 ± 11
	CD20+Int	19 ± 8***	14 ± 3*	19 ± 7***	29 ± 14*
	CD20+Bz	9 ± 3***	16 ± 3*	20 ± 10***	8 ± 3**
	CD20+Int+Bz	15 ± 5***	22 ± 4	31 ± 11**	11 ± 2**
**Spleen**					
	CD20	72 ± 35	3.12 ± 1.37	96 ± 17	46 ± 15
	CD20+Int	2 ± 0.8***	0.12 ± 0.04**	13 ± 6**	12 ± 5**
	CD20+Bz	30 ± 11*	0.73 ± 0.25**	20 ± 9**	8 ± 3**
	CD20+Int+Bz	2 ± 0.2***	0.29 ± 0.11**	5 ± 0.9***	14 ± 5**

All analysis was performed by post-hoc test ($^{*}P< 0.05$; $^{**}P< 0.01$, $^{***}P< 0.001$) and Data were shown as mean±SEM. Number of mice per group (*n*=5-6). **Abbreviations:** Bz, bortezomib; CD20, anti-mouse CD20antibody; Int, Integrin blocking antibodies, anti-LFA1 and anti-VLA4 antibodies
